# Tenecteplase Catheter-Directed Thrombolytic Therapy in Submassive Pulmonary Embolism: A Case Report

**DOI:** 10.1155/2024/3839630

**Published:** 2024-08-21

**Authors:** Dania Ghaziri, Hisham Bou Fakhreddine, Fadi Sawaya, Farah Jaber, Imad Bou Akl

**Affiliations:** ^1^ Department of Pharmacy American University of Beirut Medical Center, Beirut, Lebanon; ^2^ Department of Internal Medicine American University of Beirut Medical Center, Beirut, Lebanon

**Keywords:** catheter-directed thrombolysis, pulmonary embolism, tenecteplase

## Abstract

**Introduction:** In pulmonary embolism (PE), when used for catheter-directed thrombolysis (CDT), low-dose alteplase is associated with good outcomes. Tenecteplase has been only used as intravenous for this indication. In the context of our national economic crisis where alteplase was unavailable, we describe our experience with tenecteplase CDT.

**Case:** A 73-year-old male, hypertensive and smoker with COPD, presented to the ED with intermediate high-risk PE.(ED) with intermediate high-risk PE. Heparin infusion was initiated. A few hours later, the patient developed atrial fibrillation (AF) for which amiodarone infusion was started. Also, a left femoral and popliteal vein thrombosis was also confirmed by the lower extremity duplex. As the patient remained dyspneic with unstable vital signs, the decision was to perform a CDT. In the absence of alteplase, tenecteplase was used at 0.5 mg/h over 30 h, for a total of 15 mg.

**Result:** Twenty-four hours after tenecteplase initiation, dyspnea and vital signs had significantly improved. Oxygen support was gradually dropping to finally stop. Being on concomitant heparin infusion, the patient had a mild blood oozing at the femoral vein site of entry; however, this did not require any transfusion or discontinuation of heparin. The patient regained his baseline physical and mental functions and was discharged on enoxaparin and amiodarone tablet.

**Discussion:** This is the first experience describing the use of tenecteplase as part of CDT in a patient with acute intermediate high-risk PE. The combination to therapeutic heparin infusion, already described in different clinical scenarios with intravenous tenecteplase, was safe and well tolerated

**Conclusion:** CDT with tenecteplase was, for the first time, safely and effectively used in an intermediate high-risk PE patient. However, more studies are needed to confirm and establish these findings.

**Summary**

• Usually, at the blockage of a lung artery with a clot (a thick mass of blood elements), alteplase can be inserted via a flexible tube through a narrow opening of a vessel to dissolve this clot, whereas tenecteplase is given only directly in the vein for this same indication.
• In the context of unavailability of alteplase, we used tenecteplase via a flexible tube for a high-risk patient presenting for a blockage of his lung artery with a clot.
• Twenty-four hours after the intervention, the patient improved significantly enough to later leave back home.
• This is the first time tenecteplase is used via a flexible tube to dissolve the clot in a blocked lung artery. However, this needs to be repeated on more patients to confirm the success of the intervention.

## 1. Introduction

Patients with pulmonary embolism (PE) and hemodynamic instability have a high mortality risk. The selective use of catheter-directed thrombolysis (CDT) with low-dose alteplase [1] and low-dose anticoagulation is associated with improved outcomes, such as improved hemodynamics, and decreased right ventricular (RV) dilatation when compared to anticoagulation alone [2]. CDT with alteplase has been associated with fewer complications as compared to intravenous thrombolysis [3].

Tenecteplase, a recombinant tissue plasminogen has been used in intravenous form in patients with high-risk PE [4–6] but never investigated as part of CDT in PE. No existing literature describes and supports its use for this indication.

In the context of the Lebanese national economic crisis where alteplase was not available, we describe the case of a 73-year-old male presenting with submassive PE treated, as a first attempt described in the literature, with CDT using tenecteplase.

## 2. Case

A 73-year-old male patient, with a history of heavy smoking, hypertension, and advanced untreated chronic obstructive pulmonary disease (COPD) due to noncompliance, presented to the emergency department (ED) with a 2-day history of worsening dyspnea and dizziness. His dyspnea was severe and was accompanied by hypoxia with saturation reaching 76% when he arrived at the ED where oxygen by nasal cannula was started. First, a COPD exacerbation was ruled out. The patient was started on levofloxacin and methylprednisolone.

However, in the ED, the patient was still tachycardic (heart rate [HR] reaching 112 beats per minute) and tachypneic (respiratory rate [RR] reaching 32 breaths/minute), with blood pressure (BP) reaching 96/71 mmHg.

C-reactive protein (CRP) and D-dimer were 159 mg/L and 1925 DDU, respectively. N-terminal pro b-type natriuretic peptide (NT-proBNP) was 4470 pg/mL.

Computed tomography angiography of the chest was done and showed multiple pulmonary emboli in the right main pulmonary artery, as well as segmental and subsegmental branches of the right upper, middle, and lower lobes. It also showed advanced changes of emphysema.

Bedside echocardiography showed RV strain with dilated hypokinetic RV and moderate hypertension (PASAP = 50 mmHg). The left ventricle (LV) systolic function was normal with an LVEF of 65%–70%.

Therapeutic heparin drip was started as the patient was stratified as intermediate high-risk PE. Six hours later, the patient developed atrial fibrillation (AF), and an amiodarone intravenous drip was started. A lower extremity venous duplex showed left femoral and popliteal vein thrombosis.

The patient was transferred to the ICU for monitoring and continuation of therapy. He reverted back to sinus rhythm but continued to have sinus tachycardia with severe dyspnea and borderline BP of 92/73 mmHg. The decision was made to start him on CDT. As alteplase was not available, tenecteplase was used as an alternative.

Under ultrasound guidance, an 8F sheath was inserted into the right femoral vein. A Terumo wire was then advanced into the RV and then to the right pulmonary artery. A McNamara 4F catheter was positioned in the right pulmonary artery. Tenecteplase was then infused through the catheter at a rate of 0.5 mg/h over 30 h, for a total of 15 mg. This dose was selected in the context of his intermediate to high-risk PE to assure efficacy yet lessen the risk of bleeding.

The bags were prepared in the pharmacy, under laminar air flow by first reconstituting 50 mg of tenecteplase with 10 mL of water for injection and then by adding 1 mL (5 mg) of this solution to 499 mL of NSS. The resulting 0.01 mg/mL solution is stable for 24 h at room temperature [7]. The pharmacy prepared three bags, each with a total of 5 mg in 500 mL of NSS, to be administered over 10 h. Our patient was kept on a therapeutic heparin drip during the fibrinolytic infusion and after.

## 3. Results

The day following tenecteplase administration, our patient was comfortable with stable vital signs: median HR of 90 bpm and BP normalizing to a median of 114/39 mmHg. The oxygen support was gradually decreased from 5 L by face mask to room air. Bedside TTE showed mild improvement in RV and systolic function when compared to the findings before the tenecteplase infusion.

During the time of infusion, the patient had blood oozing at the femoral vein site of entry, and the dressing was changed. He had a drop of Hg from 13.8–12.8 g/L. No transfusion of blood was given, and the blood oozing was considered acceptable by the treating team. The decision was thus made to keep both heparin and tenecteplase.

The patient remained stable and was later discharged home on enoxaparin 1 mg/kg SC every 12 h and amiodarone 200 mg tablet daily. Two weeks after discharge, enoxaparin was switched to apixaban, and no subsequent complications were reported since then.

## 4. Discussion

To our knowledge, this is the first experience describing the use of tenecteplase as part of CDT in a patient with acute PE with intermediate to high risk, and we could not identify any similar case in the literature. The dose chosen was similar to the dose tried in a prospective nonrandomized trial for CDT tenectaplase in DVT and peripheral arterial occlusion [8]. In this study, tenecteplase was used at doses between 0.25 and 0.5 mg/h. We elected to use a high dose of 0.5 mg/h due to the higher risk of hemodynamic decompensation in case of treatment failure. To preserve the physical and bacteriological stability of our preparation, three bags of 5 mg in a total of 500 mL of NSS [7] were prepared under laminar air flow, kept in the fridge, and administered one after the other over a total of 30 h.

Bleeding was also described in this study with 7.3% as mild bleeding and 1.8% as severe [8].

Our patient had a minor hematoma at the insertion site of the catheter with a minimal decrease in Hg level not necessitating transfusion or the interruption of the continuous infusion of thrombolytics or heparin. Combining intravenous tenecteplase to heparin is well established in the literature and has been investigated in acute myocardial infarction [9], DVT, CDT [10], and submassive PE with intravenous use of both tenecteplase and therapeutic doses of heparin [6].

The patient came to the hospital 2 days after the onset of his PE and worsening respiratory symptoms because he assumed that his symptoms were related to his severe COPD. This has been described in a multicenter cross-sectional study with prospective follow-up conducted in seven French hospitals where 5.9% presenting for worsening respiratory symptoms of COPD were found to have PE [11]. In this context, CDT tenecteplase safely reversed PE manifestation in a high-risk patient.

Our experience with CDT tenecteplase in a patient with intermediate to high-risk PE was successful. This success may be attributed to the fact that our patient had no baseline bleeding risk and only hypertension as a comorbidity. However, extending this procedure to a broader patient population may be limited by the presence of higher baseline bleeding risks or the presence of multiple comorbidities.

Future research should thus involve conducting CDT with tenecteplase on a larger and more diverse patient population as well as comparing the safety and efficacy of CDT with tenecteplase versus CDT with alteplase in intermediate to high-risk PE.

## 5. Conclusion

CDT tenecteplase was, as a first attempt described in the literature, safely and effectively used in intermediate to high-risk PE. This adds the option of a life-saving intervention when other alternatives are contraindicated or unavailable. Nevertheless, more studies are needed to assess the efficacy and safety of CDT tenecteplase for intermediate high-risk PE.

## Data Availability

The datasets generated and/or analyzed during the current study are available from the corresponding author on reasonable request.
